# Early versus late initiation of renal replacement therapy in critically ill patients with acute kidney injury (The ELAIN-Trial): study protocol for a randomized controlled trial

**DOI:** 10.1186/s13063-016-1249-9

**Published:** 2016-03-18

**Authors:** Alexander Zarbock, Joachim Gerß, Hugo Van Aken, Andreea Boanta, John A. Kellum, Melanie Meersch

**Affiliations:** Department of Anesthesiology, Intensive Care and Pain Medicine, University of Münster, Albert-Schweitzer-Campus 1, Gebäude A1, 48149 Münster, Germany; Institute of Biostatistics and Clinical Research, University of Münster, Münster, Germany; Department of Critical Care Medicine, Clinical Research, Investigation, and System Modelling of Acute Illness (CRISMA) Center, University of Pittsburgh, Pittsburgh, PA USA

**Keywords:** Critically ill patients, Renal replacement therapy, Acute kidney injury, Clinical trial

## Abstract

**Background:**

Acute kidney injury remains a common complication in critically ill patients and despite multiple trials and observational studies, the optimal timing for initiation of renal replacement therapy is still unclear. The early versus late initiation of renal replacement therapy in critically ill patients with acute kidney injury (ELAIN) study is a randomized, single-center, prospective, two-arm, parallel group trial to reduce mortality in patients with severe acute kidney injury. We describe the study design and discuss aspects of the need for a trial in this patient cohort.

**Methods/design:**

Our plan is to randomize critically ill patients with acute kidney injury to ‘early’ or ‘late’ initiation of renal replacement therapy according to stage 2 and 3 of the KDIGO classification using a specific trial protocol. We plan to guide data collection and analysis using pre-existing definitions and testing. The primary endpoint is overall survival in a 90-day follow-up period. Secondary endpoints include 28-day, 60-day, 90-day and 1-year all-cause mortality, recovery of renal function, ICU and hospital length-of-stay. The primary analysis will be an intention-to-treat analysis; secondary analyses include treated analyses. We will also specify rules for handling data and determining outcome.

**Discussion:**

Several challenges for study design and execution can be seen in our trial, and it should generate results that will inform and influence the practice of renal replacement therapy in critically ill patients with acute kidney injury.

**Trial registration:**

German Clinical Trials Register: DRKS00004367 (www.germanctr.de); 28 May 2013.

**Electronic supplementary material:**

The online version of this article (doi:10.1186/s13063-016-1249-9) contains supplementary material, which is available to authorized users.

## Background

Acute kidney injury (AKI) is a well-recognized complication of critical illness with an important impact on morbidity and mortality [1, 2]. Despite substantial advances in our knowledge of the management of critically ill patients, mortality associated with AKI remains high [3–5]. Renal replacement therapy (RRT) causes a considerable escalation in the complexity of treatment, has inherent risks for adverse effects and increases cost of care for those with severe AKI. Although RRT is a key component of modern critical care, several fundamental principles, including the optimal timing of RRT initiation, remain unclear [6].

Although there has been interest in the timing of RRT initiation, a lack of consensus regarding the definition and stages of AKI, until recently, has limited progress. In addition, studies have used various arbitrary definitions for ‘early’ and ‘late’ initiation instead of using classification systems for AKI.

A retrospective study assessed the effect of timing of initiation of RRT on outcome in patients with posttraumatic AKI [7]. Serum BUN served as a surrogate marker to determine early versus late initiation of RRT. Survival was 20 % in the late group and 39 % in the early group (*p* = 0.041). Two prospective multicentre observational studies [8, 9] demonstrated higher mortality, longer hospital stay, longer duration of RRT and higher dialysis dependence in the late group. Bagshaw et al. [8] stratified early and late by median urea and serum creatinine at the time RRT was started and demonstrated no significant difference between late and early initiation of RRT using serum urea as surrogate marker (63.4 % vs. 61.4 %, *p* = 0.48). When stratified by creatinine, late RRT was associated with lower mortality (53.4 % vs. 71.4 %, *p* < 0.0001). However, for timing relative to ICU admission, late RRT was associated with higher mortality rates (72.8 % vs. 59 %, *p* <0.001). Shiao et al. [9] divided patients into early (RIFLE-0 or -Risk) and late (RIFLE-Injury or -Failure) RRT by RIFLE criteria and found significantly higher hospital mortality in the late group (74.5 % vs. 43.1 %, *p* = 0.002). A multicentre observational study analyzed data on timing of initiation of RRT based on the median BUN at the time of initiation of RRT [10]. No significant survival benefit in patients receiving early RRT could be demonstrated (survival 59 % late vs. 65 % early, *p* = 0.09). Unfortunately, these observational studies are limited to patients who had received RRT but excluded patients with AKI that had never received RRT. Recently, the Randomized Evaluation of Normal versus Augmented Level of Replacement Therapy (RENAL) study investigators published a subgroup analysis of the RENAL trial testing the effect of earlier commencement of RRT on the outcome of critically ill patients with AKI using RIFLE-Injury as surrogate marker [11]. Earlier initiation of RRT relative to RIFLE-Injury AKI did not show significant graded elevations in the risk of death with progressively delayed RRT. Recently, three meta-analyses suggest that earlier initiation of RRT in critically ill patients with AKI might be associated with a survival benefit [12–14]. However, the studies were heterogeneous and of variable quality.

All of these studies used creatinine, BUN and urine output according to the AKI classification systems to initiate RRT. However, these are functional markers and none of them can predict progression of renal injury and the need for renal replacement therapy [15, 16]. Therefore, in earlier prospective randomized trials some patients received RRT, although these patients might have recovered from AKI without RRT. Lately, research on AKI has focussed on new biomarkers for early detection of AKI and worsening of renal function, making them applicable for initiating RRT [17, 18]. Therefore, a prospective, randomized trial combining functional and damage markers is needed to provide evidence for the best timing of RRT in critically ill patients with AKI.

## Methods/design

**Fig. 1:**
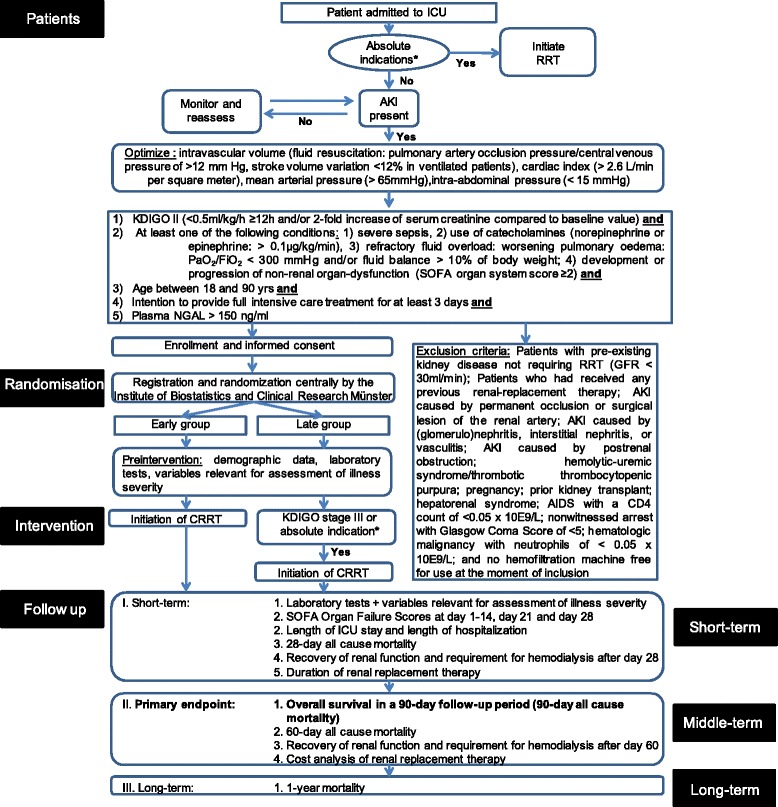
Trial workflow. Patients will be identified for recruitment by screening all patients receiving care in the ICU on a daily basis. Before enrolment, the fluid status is analyzed in cooperation with the attending physicians and optimized if necessary. After optimization is ensured, informed consent is obtained and the patient will be registered by randomization. Before initiating RRT, laboratory tests will be performed and different variables will be documented. In the ‘early’ group, RRT will be initiated immediately after randomization, whereas initiation of RRT in the ‘late’ group will be started only after reaching stage 3 of the KDIGO classification and/or if absolute indications for RRT are present. Patients randomized to ‘late’ but never reaching stage 3 will remain in the ‘late’ group for purposes of analysis. Laboratory tests will be analyzed and variables relevant for the assessment of illness severity will be recorded on day 1 to day 21, day 28, day 60, day 90 and 1 year

The early versus late initiation of renal replacement therapy in critically ill patients with acute kidney injury (ELAIN) study is a randomized, single-centre, two-arm, parallel group trial of different RRT implementation strategies for critically ill patients with AKI. Institutional review board approval was obtained from the Research Ethics Committee of the Chamber of Physicians Westfalen-Lippe and the Westfalian Wilhelms University of Münster (2012-426-f-S), Germany, and the trial is registered in the German Clinical Trials Register (DRKS00004367). The overall study flow is summarized in Fig. 1.

### Patient population

We plan to enrol 256 adult patients (age 18–90 years) fulfilling the inclusion criteria with written informed consent. If the patient is unable to provide informed consent, the legally authorized representative will be asked and in his/her absence a declaration for inclusion in an emergency situation is to be signed by a consultant physician who is not involved in the study and who is independent of the investigational team. Patient or legally authorized representative informed consent will be obtained as soon as possible. No patient will be excluded from the study on the basis of gender, race or ethnicity. Patients will be identified for recruitment by screening patients receiving care in the critical care units of our centre on a daily basis.

### Inclusion criteria

All patients are eligible if they fulfill all inclusion criteria and no exclusion criteria. The inclusion criteria are as follows:

- Kidney Disease: Improving Global Outcomes (KDIGO) stage 2 (two-fold increase in serum creatinine from baseline and/or urinary output <0.5 ml/kg/h ≥12 h) (Table 1) despite optimal resuscitation: a) optimizing intravascular volume (fluid resuscitation: pulmonary artery occlusion pressure/central venous pressure of >12 mmHg, stroke volume variation <12 % in ventilated patients); b) optimization of cardiac index (>2.6 l/min per square meter); c) hemodynamic optimization (mean arterial pressure >65 mmHg); and d) normalizing intra-abdominal pressure (<15 mmHg);

**Table 1:** Staging of acute kidney injury using KDIGO recommendations

Stage	Serum creatinine criteria	Urinary output criteria
1	Serum creatinine x 1.5 or serum creatinine rise of 0.3 mg/dl in 48 h	<0.5 ml/kg/h for 6 h
2	Serum creatinine x 2	<0.5 ml/kg/h for 12 h
3	Serum creatinine x 3 or serum creatinine ≥4 mg/dl or renal replacement therapy	<0.3 ml/kg/h for 24 h or anuria for 12 h

KDIGO, Kidney Disease: Improving Global Outcomes [6]
- Plasma neutrophil gelatinase-associated lipocalin (NGAL) >150 ng/ml;
- At least one of the following conditions: a) severe sepsis; b) use of catecholamines (norepinephrine or epinephrine >0.1 μg/kg/min); c) refractory fluid overload (worsening pulmonary edema, PaO2/FiO2 < 300 mmHg and/or fluid balance >10 % of body weight); and d) development or progression of non-renal organ dysfunction (Sequential Organ Failure Assessment (SOFA) score ≥2);
- Age between 18 and 90 years; and
- Intention to provide full intensive care treatment for at least 3 days.

### Exclusion criteria

The exclusion criteria are as follows:

- Pre-existing chronic kidney disease not requiring RRT (GFR <30 ml/min);
- Previous RRT;
- AKI caused by permanent occlusion or surgical lesion of the renal artery;
- AKI caused by glomerulonephritis, interstitial nephritis or vasculitis;
- AKI caused by postrenal obstruction;
- Hemolytic-uremic syndrome/thrombotic thrombocytopenic purpura;
- Pregnancy;
- Prior kidney transplantation;
- Hepatorenal syndrome;
- AIDS with a CD4 count of <0.05 x 10E/l;
- Hematologic malignancy with neutrophils of <0.05 x 10E/l;
- No hemofiltration machine free for use at the moment of inclusion; and
- Participation in another interventional clinical trial.

### Enrolment

Enrolment began in August 2013. Prior to being randomized into the study, the trial coordinators obtain consent for participation in the study (see Ethics and consent). Assuming all inclusion criteria are fulfilled and no exclusion criteria are met, each patient receives a study identification number and treatment allocation at enrolment. Patients are randomized in a 1:1 ratio to one of the two treatment arms using a computerized system. Randomization is stratified by SOFA Cardiovascular organ Failure Score (0–2 vs. 3–4) and by the presence or absence of oliguria. The randomization day is day 0.

### Treatment arms

Early initiation of RRT will be initiated at stage 2 of the KDIGO classification within 8 hours:

- Urine output <0.5 ml/kg/h for ≥12 h; and/or
- Two-fold increase of the serum creatinine level compared to the baseline value.

Late initiation of RRT will be initiated when stage 3 of the KDIGO classification (not later than 12 hours after achieving stage 3) is achieved:

- Urine output <0.3 ml/kg/h for ≥24 h; and/or
-  > Three-fold increase of the serum creatinine level compared to the baseline value; and/or
- Serum creatinine of ≥4 mg/dl with an acute increase of at least 0.5 mg/dl within 48 hours.

Or if any of the following absolute indications for RRT are present:

- Urea serum levels >100 mg/dl;
- Potassium serum levels >6 mmol/l and/or ECG abnormalities;
- Magnesium serum levels >4 mmol/l;
- Urine production <200 ml/12 h or anuria (without diuretics, according to the KDIGO recommendations); and
- Organ edema in the presence of AKI resistant to diuretic treatment (one attempt with loop diuretics prior to randomization).

After enrolment, ELAIN investigators are responsible for the catheter insertion and the initiation of RRT (see Additional file 1). Daily visits will be performed to ensure adherence to the protocol. Hemodynamic, pulmonary, inflammation, renal and laboratory data will be documented. Education to ensure proper implementation of the study protocol was done prior to starting enrolment. Project manager and trial investigators conduct monthly conferences to monitor all aspects of the protocol.

### Patient management

All patients receive standard intensive care therapy. As no pharmacological therapy for AKI exists, the management of AKI remains primarily supportive, with renal replacement therapy serving as a cornerstone of therapy for patients with severe kidney injury. None of the patients in both groups (‘early’ and ‘late’ groups) are exposed to additional risks.

The patient’s primary physicians determine the remainder of patient management, consistent with established best practices for the management of critically ill patients (e.g. use of diuretics, application of fluids). Measuring central venous pressure and cardiac output is performed in all patients. These co-interventions are not standardized, but they are recorded and will be analyzed after finishing the trail. As this is a prospective randomized trial, there should not be any differences between the groups. Further treatment rules are described in Additional file 1.

### Outcomes

The primary endpoint is overall survival in a 90-day follow-up period. If the difference in overall survival is significant, the treatment effect will be estimated by means of the 90-day all-cause mortality rate in both treatment groups.

Secondary outcomes include:

- Overall survival in a 28-day and 60-day follow-up period;
- Overall survival in a 1-year follow-up period;
- Clinical evidence of organ dysfunction (daily SOFA scores while in the ICU);
- Recovery of renal function as defined in the KDIGO guidelines [6];
- Requirement of haemodialysis after day 28 and day 60;
- Duration of renal support;
- ICU and hospital length of stay; and
- Markers of inflammation, oxidative stress, cellular hypoxia and coagulation.

### Ethics and consent

The present trial protocol is performed in accordance with the Declaration of Helsinki (version October 2008; 49th General Assembly of the World Medical Association, Somerset West, Republic of South Africa) and approved by the institutional review board from the Research Ethics Committee of the Chamber of Physicians Westfalen-Lippe and the Westfalian Wilhelms University of Münster.

Data collection will be performed pseudonymously and the patient’s name will not appear on any case report form (CRF) or in any other trial document. All data will be kept confidential. Consent procedures follow local requirements, as approved by the ethics committee of the University of Münster. The treating investigator will inform the patient about the nature of the trial, its aims, expected advantages as well as possible risks. Written informed consent will be obtained from eligible patients and in case of patient incapacity by the legally authorized representative (see Additional file 2). In case of an emergency situation a consultant physician who is independent of the investigational team will be involved. Once the participant regains capacity or the legally authorized representative is available, he/she will be asked to affirm or withdraw consent.

### Objectives and aims

#### Aim 1

To compare the clinical efficacy between early and late initiation of renal replacement therapy in critically ill patients with AKI, we are testing the following:

- Hypothesis I: early initiation of renal replacement therapy in the treatment of critically ill patients with AKI will reduce mortality at 90 days compared to late initiation.

#### Aim 2

To understand the mechanisms of illness and recovery and how the RRT implementation strategies affect them, we are testing the following:

- Hypothesis II: early and late RRT are associated with differences in the expression of markers of illness and recovery.

#### Aim 3

We aim to assess the costs and cost-effectiveness of both strategies.

### Statistical analysis

Statistical analyses will be performed according to the principles of the ICH guideline E9 ‘Statistical principles for clinical trials’ [19] using standard statistical software (IBM SPSS Statistics, Armonk, NY, USA).

### Power and sample size

Power calculations are performed based on the primary endpoint, i.e. the overall survival in a 90-day follow-up period. The primary efficacy analysis is intended to test whether early versus late initiation of RRT in intensive care patients with AKI results in different overall survival in a 90-day follow-up period.

An adaptive design based on a group sequential plan according to O’Brien and Fleming with one interim analysis and a global (two-sided) significance level α = 0.05 is applied (see below). The expected 90-day mortality rate in the control group with late initiation of RRT is 55 % (based on literature research [7–10, 20–24]). Differences between treatment groups are to be detected with a power of 80 %, if the 90-day mortality rate in the experimental intervention group with early initiation of RRT is 37 % or smaller. The expected treatment effect of 18 % is calculated on the mortality differences between the early and late groups resulting from the studies of our literature research [7–10, 20–24] (see Additional file 3). The interim analysis is conducted at the time when half of the total number of deaths has been observed (information rate 0.5). Follow-up of each patient will be 90 days. Resulting from these considerations, the interim analysis is performed after 53 deaths have been observed across both treatment groups. The final analysis is planned to be performed after 106 deaths have been observed in total across both treatment groups, i.e. assuming an average of 46 % mortality rate in the 90-day follow-up period, a total number of 106/0.46 = 230 patients across both treatment groups is included in the final analysis. This corresponds to 256 recruited patients, if an expected number of 10 % of recruited patients is assumed to be lost to follow-up and in the worst case has completely non-evaluable data. Power calculations are to be performed based on a two-sided inverse normal log-rank test [25], using ADDPLAN software (ICON, Dublin, Ireland).

### Interim analysis

A group sequential plan according to O’Brien and Fleming with one interim analysis is established. In order to maintain a global significance level alpha = 0.05, the interim and the final analysis are performed on local significance levels of 0.0052 and 0.0480, respectively. Based on the group sequential plan, an adaptive design is applied, that admits two possible design changes after the interim analysis: i) the number of deaths of the final analysis will be re-calculated applying the inverse normal method [26]; and ii) the schedule and the number of further interim analyses may be modified applying the conditional rejection error probability (CREP) principle [27, 28].

#### i) Re-calculation of the number of deaths of the final analysis applying the inverse normal method

The basic idea of the inverse normal method is to transform the *p* values of successive stages of a multistage trial to normally distributed test statistics that can be processed using group sequential methods. In particular, let *p*1 and *p*2 denote the *p* values of the interim and final statistical analysis, respectively. Then in the final analysis the null hypothesis is rejected if:

$$ C\left(p1,p2\right):=1-\varnothing \left[\sqrt{\frac{1}{2}}\kern0.5em \cdotp \kern0.5em {\varnothing}^{-1}\left(1-p1\right)+\sqrt{\frac{1}{2}}\kern0.5em \cdotp \kern0.5em {\varnothing}^{-1}\left(1-p2\right)\right]\le {\alpha}_c. $$

The term *α*_*c*_ is determined so that the overall type I error is controlled.

Interim data on the primary outcome comprise the treatment arm, the date of randomization, a status indicator of death within 90 days post-randomization, as well as the data of death or last follow-up. Using these data the conditional power of a significant final result is calculated: a) based on the observed hazard ratio of early versus late initiation of RRT at the time of the interim analysis; as well as b) based on the originally assumed hazard ratio. The number of deaths of the final analysis is determined as follows:

- If the originally planned number of deaths of the final analysis yields a conditional power larger than (1 − *β*_*upper*_^*cond*^) calculated according to both approaches a) and b), then the originally planned number of deaths is kept unchanged.
- If in one or both of the two approaches a) and b) the conditional power is between (1 − *β*_*lower*_^*cond*^) and (1 − *β*_*upper*_^*cond*^), then the number of deaths of the final analysis is increased, so that in both approaches a) and b) the conditional power is at least equal to (1 − *β*_*upper*_^*cond*^). In any case, however, the maximum number of additionally recruited patients in the second stage of the trial is restricted to an upper limit of 350 patients.
- If in both approaches a) and b) the conditional power is lower than (1 − *β*_*lower*_^*cond*^), then no further patient is recruited and the study is stopped.

The values (1 − *β*_*lower*_^*cond*^) and (1 − *β*_*upper*_^*cond*^) are kept confidential in order to preserve the integrity of the trial. Otherwise, if the number of additionally recruited patients in the second stage of the trial is announced, this information could be used to disclose the results of the interim analysis.

#### ii) Modification of the schedule and the number of further interim analyses applying the CREP principle

Upon inspection of interim results beyond the re-calculation of the number of deaths, further possible design changes will be performed, i.e. a modification of the schedule and the number of further interim analyses. These changes will be done according to the CREP method [27, 28]. The CREP principle is a general method used in adaptive clinical trials. In an interim analysis based on current data, the CREP is calculated, that is the conditional probability that in the final analysis a significant result will be reached, under the initial study design, and under the null hypothesis, conditional on the information that is available so far. Denote this probability CREP_0_. The CREP principle then suggests that after the interim analysis a completely different design may be chosen – if the new design is constructed so that it provides the same conditional rejection error probability CREP_0_ as the initial design.

Modifications to the protocol will be made only in the form of written amendments and with the agreement of the study committee. The respective ethics committees will be informed of the modifications and patient information will be changed according to the modifications of the protocol.

Suppose, for example, that upon the inspection of interim results according to i) it is decided to continue to follow-up patients and to perform the final analysis at the time when 151 deaths have been observed in total across both treatment groups. Moreover, suppose that the calculated conditional rejection error probability at the time of the interim analysis amounts CREP_0_ = 0.04. In this case it may be decided to introduce another interim analysis before the final analysis is performed. Therefore, a new group sequential plan is established and implemented at the time of the (first) interim analysis. The new group sequential plan consists of one (further) interim analysis before the final analysis is performed after 151 deaths have been observed, and maintains a (two-sided) significance level alpha = CREP_0_ = 0.04. Concretely, the new group sequential plan may be established according to Pocock, so that the second interim analysis is performed after 53 + 49 = 102 deaths have been observed in total, and both the second interim and the final analysis are performed on local significance levels of 0.0233, respectively.

### Primary outcome analyses

**Fig. 2:**
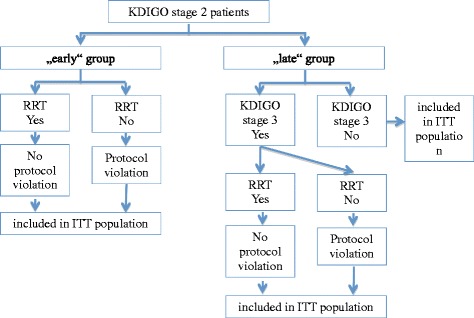
Statistical analysis with intention-to-treat principle. All recruited ELAIN patients randomly allocated to the early or late group will be considered for intention-to-treat. Patients with protocol violation will be included in the analysis to gain a representative result of the daily practice. Protocol violation is defined as: subjects in the early group who did not receive RRT in the early phase of AKI (KDIGO stage 2); and subjects in the late group who did not receive RRT, although KDIGO stage 3 was achieved and patients were lost to follow-up

The primary efficacy analysis will include all randomized patients (full analysis set) and will be performed according to the intention-to-treat principle, i.e. all patients are analyzed in the group to which they were randomized (Fig. 2). The effect of early initiation of RRT versus late initiation of RRT on overall survival in a 90-day follow-up period will be assessed by comparing the randomized groups with a (two-sided) inverse normal log-rank test [21]. If the difference in overall survival is significant, the treatment effect will be estimated by means of the 90-day all-cause mortality rate in both treatment groups. The primary intention-to-treat analysis of the primary outcome provides confirmatory statistical evidence.

**Fig. 3:**
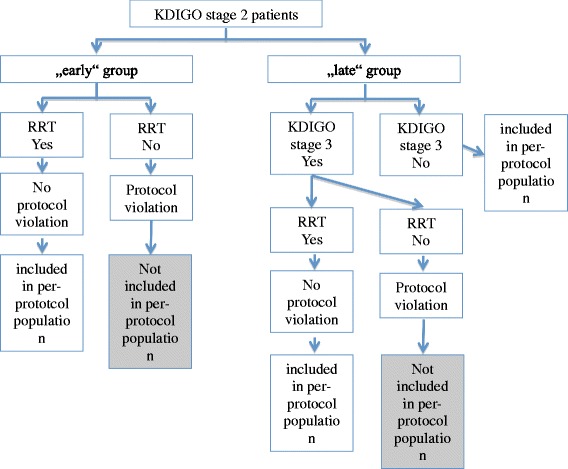
Statistical analysis with per-protocol principle. Patients with protocol violation will be excluded in the analysis to analyze the effect of both treatment arms. Protocol violation is defined as: subjects in the early group who did not receive RRT in the early phase of AKI (KDIGO stage 2); and subjects in the late group who did not receive RRT, although KDIGO stage 3 was achieved

Beyond the above primary analysis, the following pre-specified sensitivity analyses will be performed (see Fig. 2). In per-protocol analyses only patients without major protocol violations are included (see Fig. 3); in particular, patients are included only if they complete 90-day follow-up (complete case analysis). Using these data two kinds of analysis are performed in order to address two different questions.

- In a first approach, the primary outcome is evaluated (overall survival in a 90-day follow-up period) using a two-sided inverse normal log-rank test [21]. The only difference to the primary analysis is that a per-protocol approach is pursued instead of an intention-to-treat analysis.
- In a second approach, data are reduced from the original primary outcome to the binary outcome ‘90-day mortality from all causes’. The binary outcome is created in order to provide comparability to other published study results. It is evaluated using a two-sided Chi-squared test.

Further statistical analyses of the primary outcome will be performed in order to address the fact that study patients have different risk profiles at the time of RRT initiation. The efficacy of the experimental and the control treatment may differ depending on the patient’s risk profile. For example ‘high risk’ patients may benefit from experimental treatment with early initiation of RRT substantially compared to control treatment, whereas in ‘low risk’ patients the ‘late’ approach may be superior. In order to address these questions, multivariable statistical analyses will be performed using Cox regression. Overall survival is modelled as a function of baseline risk (determined by pre-randomization baseline data such as the patient’s age, Acute Physiology and Chronic Health Evaluation (APACHE) score and SOFA score, as well as the baseline medical condition, i.e. severe sepsis/use of catecholamines/refractory fluid overload/development or progression of non-renal organ dysfunction), RRT treatment and the interaction of baseline risk and RRT treatment. In three different model approaches, RRT treatment is expressed in terms of: i) a time-dependent covariate that impacts survival starting with the time of RRT initiation; ii) the randomized treatment group; and iii) the time to initiation of RRT.

### Secondary and tertiary outcome analyses

Statistical analysis of the pre-specified secondary endpoints will be performed with descriptive and inductive statistical methods. Survival data will be described by Kaplan–Meier plots and survival distributions will be compared using the log-rank test. Ordinal scores and non-normally distributed metric measures will be analyzed using non-parametric methods, including calculation of the median and quartiles and comparison of distributions using the Mann–Whitney U test. In case of normally distributed metric measures parametric methods will be used, including calculation of the mean and standard deviation and comparison of distributions using Student’s t-test. Binary data will be presented by contingency tables and rates will be compared using the Chi-squared test. Secondary and tertiary outcome analyses are considered not to be confirmatory. The *p* values are regarded noticeable in case *p* ≤0.05. No adjustment for multiple testing will be performed. Therefore, an overall significance level is not determined and cannot be calculated.

Additional exploratory analyses will include model-based analyses, subgroup analyses and safety analyses. In safety analyses, all study patients will be included (safety population).

### Trial management

#### Protocol adherence

Initiation and close-out visits will be performed. The trial site will be regularly visited during recruitment and follow-up. The scope of these visits is to check compliance of the trial site with the study protocol and ‘good clinical practice’ rules. For all patients, the informed consent documents will be checked. In addition, source data verification of the key data (eligibility criteria, intervention, outcome measures) will be performed routinely in a random sample of 50 % of the patients. Monitoring will follow the standard operating procedure (SOP) based on the Technologie- und Methodenplattform für die vernetzte medizinische Forschung e.V. (TMF)-SOPs.

### Data collection and management

All trial-related processes will follow the SOPs. According to the SOPs, key documents and processes are subject to internal review. The data will be collected on paper CRFs and stored at the Department of Anesthesiology, Intensive Care and Pain Medicine at the University of Münster. Monitoring will include a timely query management process based on consistency and plausibility checks, combined with a dunning process for missing documentation.

### Safety

The Executive Committee is the management and decision-making body for the operational aspects of the study and will monitor the performance of the medical center and the quality of data collected. The Executive Committee will formulate publication plans and will oversee the publication and presentation of all data from the study. The Committee must grant permission before any study data may be used for presentation or publication.

## Discussion

The decision to start RRT in critically ill patients with severe AKI can depend on numerous factors and is, therefore, a complex process [8]. Timing of RRT has been difficult to study and has shown considerable variation between clinicians and institutions [29]. As a consequence, the question of the optimal timing of the initiation of RRT has not been solved yet [8–11, 13, 14]. Theoretically, initiation of RRT before the onset of severe AKI might influence patient outcomes. Specifically, early RRT might attenuate kidney-specific and non-kidney organ injury from acidemia, uremia, fluid overload and systemic inflammation, and potentially translate into improved survival and earlier recovery of kidney function [17, 18]. The results of three meta-analyses already showed that the early initiation of RRT might have a potential survival benefit for critically ill patients with AKI [12–14]. However, on the contrary, early initiation of RRT might involve patients who would recover renal function with conservative treatment alone. Consequently, this would result in unnecessary RRT with potential risks associated with RRT, raising an important ethical issue.

The ELAIN trial is a prospective randomized trial with the objective to analyze the influence of RRT timing on survival of critically ill patients with AKI. There are some aspects of the trial protocol that bear specific mention.

The study design contains a specific protocol that tends to minimize unnecessary RRT trying to prevent patients from avoidable RRT-related risks and improve patients’ outcome. In 2012, the KDIGO group published the KDIGO criteria for diagnosing AKI [6]. It has been shown that only 20 % of patients who develop KDIGO stage 2 progress to KDIGO stage 3 [30]. Sepsis and other severe conditions (e.g. hemodynamic instability manifested by hypotension requiring vasopressor support, refractory fluid overload) have been shown to be important risk factors for developing dialysis-dependent AKI [31, 32]. Therefore, the inclusion criteria chosen for the ELAIN trial were selected to identify patients who are at high risk for the need of RRT. In addition, we chose a biomarker-based model for the detection of patients with worsening AKI, because previous studies demonstrated that biomarkers can be used for identifying potential aggravation [16]. By using this patient-enrichment strategy, we excluded patients whom subsequently recovered from AKI spontaneously without requiring RRT. Plasma NGAL is one of the most studied biomarkers in the field of AKI. The diagnostic value for the detection of AKI might be controversial [33, 34] but for the need of RRT in critically ill patients it has been demonstrated as a good predictor [35, 36]. Thus, to reduce the risk of unnecessary RRT, we only include patients with KDIGO stage 2, concomitant disorders and elevated NGAL values [35].

Nevertheless, the ELAIN trial has some limitations. The power calculation is based on data published a long time ago. Although the mortality of dialysis-dependent AKI decreased over the last 20 years [37], the mortality is still unacceptably high. A recently published meta-analysis regarding the initiation of renal replacement therapy analyzed 15 trials published between 1985 and 2011 and the authors showed that the overall 28-day mortality across the 15 trials was 55 % [13]. Although this study included older studies, a recently published trial investigating critically ill patients with a dialysis-dependent AKI also demonstrated that the 60-day all-cause mortality in critically ill patients with a dialysis-dependent AKI is 52 % [38]. Another limitation of the study is that we include critically ill patients with AKI stratified by hemodynamic SOFA score and urinary output accepting heterogeneity due to the inclusion of septic and non-septic patients. The pathophysiology of septic AKI is unique resulting in differences in clinical outcomes and responses to renal replacement therapy compared to non-septic patients [31]. But implementing a specific protocol with homogenous criteria for the diagnosis of critical illness and acute kidney injury combined with biomarkers is the first step to filling an important knowledge gap. Another limitation of such a trial design is that the investigators cannot be blinded and consequently there is a risk of contamination or unequal prescription of co-interventions. However, we adequately take this into account by a clear monitoring of all interventions.

Several studies investigating the timing of continuous RRT in critically ill patients used the time from randomization to commencement of continuous RRT to define ‘early’ and ‘late’ [8, 39]. In our trial we use the severity of AKI based on the KDIGO criteria to define ‘early’ and ‘late’ initiation of continuous RRT. The initiation of RRT represents an escalation in the level of care and is associated with certain risks. In the past, it has been shown that the Acute Kidney Injury Network (AKIN) criteria alone cannot predict the progression of the severity of AKI [30]. But the biomarker NGAL has been shown to predict the progression of the severity of AKI [40, 41] and the need for RRT in critically ill patients [15, 35]. Therefore, we combine the KDIGO criteria with clinical risk factors and NGAL to increase the probability that we only include patients with severe AKI who subsequently need RRT and exclude patients who spontaneously recover from AKI. This might not be a validated approach, but the study results will show whether the combination of these factors can identify patients who subsequently develop a dialysis-dependent AKI.

The ELAIN trial is the first randomized controlled trial that includes a biomarker in a specific protocol plan to detect patients who will develop a severe, dialysis-dependent AKI. The results might be a significant contribution to the therapy of critically ill patients with acute kidney injury.

### Trial status

Recruitment is active.

## Additional files

Additional file 1: Treatment rules for the ELAIN trial. (DOCX 122 kb) Additional file 2: Informed consent form for the ELAIN trial (unapproved translation). (PDF 396 kb) Additional file 3: Literature overview for the design of the ELAIN trial. (PDF 68 kb)

## Abbreviations

AKI: acute kidney injury
AKIN: Acute Kidney Injury Network
APACHE: Acute Physiology and Chronic Health Evaluation
BUN: blood urea nitrogen
CREP: conditional rejection error probability
CRF: case report form
ECG: electrocardiogram
ELAIN: early versus late initiation of renal replacement therapy in critically ill patients with acute kidney injury
GFR: glomerular filtration rate
ICU: intensive care unit
KDIGO: Kidney Disease: Improving Global Outcomes
NGAL: neutrophil gelatinase-associated lipocalin
RRT: renal replacement therapy
SOFA: Sequential Organ Failure Assessment
SOP: standard operating procedure.
