# Erratum to: ‘Early versus late initiation of renal replacement therapy in critically ill patients with acute kidney injury (The ELAIN-Trial): study protocol for a randomized controlled trial’

**DOI:** 10.1186/s13063-016-1386-1

**Published:** 2016-05-23

**Authors:** Alexander Zarbock, Joachim Gerß, Hugo Van Aken, Andreea Boanta, John A. Kellum, Melanie Meersch

**Affiliations:** Department of Anesthesiology, Intensive Care and Pain Medicine, University of Münster, 48149 Münster, Germany; Institute of Biostatistics and Clinical Research, University of Münster, 48149 Münster, Germany; Department of Critical Care Medicine, Clinical Research, Investigation, and System Modelling of Acute Illness (CRISMA) Center, University of Pittsburgh, Pittsburgh, 48149 United States

Unfortunately, the original version of this article [1] contained an error. There is a typo in one of the formulas in the PDF version of this article on page six, though the HTML version has the correct formula. To avoid confusion the correct formula has been included here.

$$ C\left(p1,p2\right)\kern0.24em :=1-\phi \left[\sqrt{\frac{1}{2}}\cdot {\phi}^{-1}\left(1-p1\right)+\sqrt{\frac{1}{2}\cdot}\;{\phi}^{-1}\left(1-p2\right)\right]\le {\alpha}_c $$
