# Supplementary material for: Early versus late initiation of renal replacement therapy in critically ill patients with acute kidney injury (The ELAIN-Trial): study protocol for a randomized controlled trial
Source: Trials. 2016 Mar 18;17:148. doi: 10.1186/s13063-016-1249-9 (PMC4797166; doi:10.1186/s13063-016-1249-9)
Supplement: Additional file 1: — Treatment rules for the ELAIN trial. (DOCX 122 kb) [file 13063_2016_1249_MOESM1_ESM.docx]

**Appendix. Treatment rules for the ELAIN Trial**

**Rules for renal replacement therapy**

- In order to ensure uniformity of treatment between the early and the late group, it is critical that specific protocols for the performance of renal replacement therapy be strictly adhered to.
- Modality: All patients in both groups will be treated using continuous venovenous hemodiafiltration (CVVHDF). Replacement fluid will be delivered into extracorporal circuit before the filter (i.e. predilution), with a ratio of dialysate to replacement fluid of 1:1.
- Dose: The effluent flow prescribed will be based on the patient’s body weight at the time of randomization and will be 30ml/kg/h [[27](#_ENREF_27),[28](#_ENREF_28)]. Blood flow will be kept above 110 ml/min. Fluid will be removed by decreasing the flow of the replacement fluid and of the dialysate in equal proportion. The delivered dose of RRT will be monitored based on blood-side urea kinetics.
- Anticoagulation: Regional anticoagulation with citrate will be used to prevent circuit clotting.
- Cessation of RRT: RRT will be discontinued if renal recovery defined by urine output (>400ml/24h without and 2100 ml/24h with diuretic treatment) and creatinine clearance (>20ml/min) occurs.
- If cessation criteria are not fulfilled after 7 days, continuous renal replacement therapy can be changed to a discontinuous procedure (SLEDD, SCUF or IHD).

**Rules for additional treatments**

- In patients with acute lung injury or acute respiratory distress syndrome, tidal volume for mechanical ventilation will be approximately 6 ml/kg of predicted body weight and adjusted to maintain a peak plateau pressure between 25 and 30 cm of water (NEJM 2000; 342:1301-8).
- Ventilator associated pneumonia will be evaluated and treated in accordance with published clinical practice guidelines and consensus statements (AJRCCM 1996; 153:1711).
- All medications will be dose adjusted for renal failure and renal replacement therapy in accordance to standard dosing guidelines.
- Management of aspects of care that are thought to have a specific impact on outcomes in AKI (e.g., nutrition) have been specified. Management of other aspects of care for which there is consensus regarding optimal management of critically ill patients (e.g., ventilator management in ALI/ARDS, diagnosis and management of ventilator-associated pneumonia and, diagnosis and management of sepsis) will be provided in accordance with theses standards of care.
- All patients will be prescribed a nutritional intake that will provide at least 25-30 kcal/kg/d, depending on mechanical ventilation and other factors. Protein intake will be at least 1.2 g/kg/d. In patients receiving parenteral nutrition, carbohydrate infusion rates will not exceed 5 mg/kg/min. Water-soluble vitamins will be supplemented to replace dialysis-related losses.
