# Supplementary material for: Early versus late initiation of renal replacement therapy in critically ill patients with acute kidney injury (The ELAIN-Trial): study protocol for a randomized controlled trial
Source: Trials. 2016 Mar 18;17:148. doi: 10.1186/s13063-016-1249-9 (PMC4797166; doi:10.1186/s13063-016-1249-9)
Supplement: Additional file 2: — Informed consent form for the ELAIN trial (unapproved translation). (PDF 396 kb) [file 13063_2016_1249_MOESM2_ESM.pdf]

WESTFÄLISCHE WILHELMS-UNIVERSITÄT MÜNSTER

UKM Universitätsklinikum Münster

Patient information

Prospective, randomized, clinical study for analyzing the influence oft he timing of renal replacement therapy on the survival of critically ill patients with acute kidney injury (ELAIN-Trial)

Responsible institution:
Department of Anesthesiology, Intensive Care and Pain Medicine
University of Münster
Albert-Schweitzer-Campus 1, A1
48149 Münster

Principal Investigator: Prof. Dr. med. Alexander Zarbock

Dear patient,

We would like to ask you if you are willing to participate in our study called „Early versus late initiation of renal replacement therapy in critically ill patients with acute kidney injury".

The function of the kidney is the removal of metabolic end products, so called uremic substances, and toxic agents through the urine. The kidneys control the fluid balance and regulate the electrolyte and acid-base status. Several severe disorders can lead to a loss of kidney tissue and subsequently to a loss of function.

Acute kidney injury is a sudden decline of the kidneys function that may occur within a few hours till days and is generally reversible. It may appear in patients with previously healthy kidneys or in patients with chronic kidney disease. Different causes, such as sepsis, hypovolemia, shock, or adverse drug reactions (e.g. contrast agents) can lead to acute kidney injury. The consequences are dangerous levels of fluid and waste products (creatinine and urea), electrolyte and acid-base imbalances. This may result in potentially life-threatening conditions. It has been shown that acute kidney injury is correlated with adverse prognosis.

The injury of the kidney with the need for renal replacement therapy is a common complication of critical illness and, despite optimized therapeutic methods, associated with high mortality rates. To this date, the optimal time for the initiation of renal replacement therapy is unknown. In this study we want to examine whether the early initiation of renal replacement therapy is associated with a survival benefit compared to late initiation. This study is necessary to improve the treatment of critically ill patients with acute kidney injury. With the results of this research we hope to contribute to the improvement of the prognosis of patients with acute kidney injury.

The responsibility for this study is beard by the competent institution. The institution is also responsible for the compliance of legal requirements and for the implementation of clinical examinations. The prin-

cipal investigator is Prof. Dr. Alexander Zarbock (Department of Anesthesiology, Intensive Care and Pain Medicine, University of Münster).

Information

Why do we perform this study?
We perform this study to evaluate the optimal timing for the initiation of renal replacement therapy in critically ill patients with acute kidney injury.

What treatment do I receive?
We want to examine whether the early initiation of renal replacement therapy shows a benefit comparing to late initiation. If we diagnose an acute kidney injury and you are willing to participate in this study you will be assigned to one of the two treatment arms. Renal replacement therapy will be initiated either early or late.

How is the study process and what do I have to consider if I participate in this study?
At the time of study inclusion we will evaluate your medical history and you will get a comprehensive medical examination. Acute kidney injury is diagnosed through several clinical parameters such as an increase in serum-creatinine in the blood and/or a decrease in urinary output. Additionally, we will analyze a biomarker (NGAL; neutrophil gelatinase-associated lipocalin) in the blood which gives us a more detailed specification on acute kidney injury. The clinical condition with regard to acute kidney injury needs to be considered due to our inclusion criteria. It depends on the results of this comprehensive examination whether your clinical condition allows us to include you to the study. Subsequently, if you give your consent, you will be randomly assigned to one of the two treatment arms. If you are randomized to the early group renal replacement therapy will be initiated as soon as possible. If you are randomized to the late group the timing of initiation of renal replacement therapy cannot be appointed. The course of acute kidney injury depends on numerous individual factors in critically ill patients. The time interval until fulfilling the criteria for the late group can differ between hours and days.

If you participate in this study we will collect blood (3 ml) and urine (3 ml) samples at five different time points (at the time of inclusion, day 1, 3, 5 of renal replacement therapy and 1 day after cessation of renal replacement therapy) for additional biomarker analysis. Samples will be collected using routinely placed catheters, additional vascular punctures are not necessary.

Clinical examinations, medical interventions and ascertained parameters are fixed components of your routinely treatment. The intensive care treatment does not differ between the two groups except the time of the initiation of renal replacement therapy. One year after inclusion we will contact you to ask for your physical condition.

Care of samples
The samples for the additional analyses will be labeled pseudonymously. Pseudonymous means that we do not use your name or your initials for the labeling. We will use a number code for the samples.
After the analyses, which will be performed in our own laboratories, we will keep the samples for a maximum of ten years and then destroy them.

Do I have a personal benefit by participating in this study?
If you are allocated to the early group, this may improve the treatment of your disease. As it is not proved whether the early initiation of renal replacement therapy is associated with a clinical advantage, the participation in this study may not have the desired benefit.
If you are allocated to the late group, your prospects for treatment will not differ between participation and no participation in the study.
However, the results of this study may contribute to the improvement of the therapy of critically ill patients with acute kidney injury.

What risks are associated with the participation in the study?
With this study we want to analyze whether the early initiation of renal replacement therapy is associated with a clinical benefit in critically ill patients with acute kidney injury compared to late initiation. Renal replacement therapy will be performed using Citrate anticoagulation, a widely accepted and used method. For this, a double lumen venous catheter needs to be inserted and blood is extracted through one of the two lumen. The extracted blood runs through dialysis, the uremic agents are removed and brought back to the body through the second lumen. As the blood runs through extraneous surface, anticoagulation with citrate is performed to avoid clotting. The effect of citrate is reversed by giving calcium before entering the systemic circulation in order to avoid adverse events.
The following complications may occur: catheter insertion problems (bleeding, infection, tissue injury), blood pressure drops caused by blood extraction, electrolyte imbalances. All these possible complications are considered low as intensive care treatment means close monitoring of the hemodynamic and laboratory parameters and quick response to the mentioned conditions.
It is expected however that patients in both groups will need dialysis; the only difference is the timing of initiation of renal replacement therapy. For most patients, the participation in this study will not come with extra risks.

What happens if I do not participate in this study?
If you do not participate in this study you receive the routinely medical care according to the current medical standards.
Important: You are able to terminate your study participation at any time point during the study period without disadvantages for your medical treatment.

Who may participate in this study?
You are not allowed to participate in this study if you are currently participating or did participate until recently (30 days) in another trial.
Further, you are not able to participate in this study if you meet one of the following criteria:

- Pregnancy

- Chronic kidney disease (glomerulonephritis, interstitial nephritis, vasculitis)
- Previous renal replacement therapy
- Liver cirrhosis
- HIV-infection
- Hematologic diseases

The study investigator will proof the exclusion criteria and answer your questions.

Who decides whether I get eliminated from this study?
You can terminate your study participation at any time point during the study period without any explanation. This will not lead to any disadvantages in your medical treatment.
In certain circumstances the study investigator will decide to terminate your study participation and you will not have any influence on this decision. This may be caused by the following:

- Your study participation is medically unjustifying.
- The complete trial is cancelled.

Do I get information about new findings during the study?
If we have any new findings that may be related to a change in your personal therapeutic strategy then you obviously will get information on that.

Does the study participation arise any costs?
No, there are no additional costs resulting from study participation.

Am I medically insured?
Yes, we have a health insurance for study participants. By request, you can get the scope of the insurance cover.

**Name and address of the insurance:**	**Zurich Insurance plc NFD**<br>**53287 Bonn**
**Telephone:**	**0228/2682650**
**Fax:**	**0228/265666**
**Number of insurance:**	**800.540.195.001**

Any health impairment that may be related to the study participation needs to be reported immediately to the insurance to protect your insurance cover. The study investigator needs to be informed immediately about any change in your medical condition. He will evaluate if this change may be related to the study and he will help you with the report to the insurance. The status will be documented and clarified. This is of high interest because otherwise you may loose your insurance cover.
By request you will obviously receive a copy of the insurance conditions.

What happens to the data?
Your medical history and personal information will be documented pseudonymously. We will use a number code for your data to keep them safe and only staff members working on this trial have access to this code. The staff is obligated to maintain confidentiality. A decoding is only allowed on the legally prescribed cases.
Details can be seen in the informed consent.

If you have any questions
Please contact the study investigators: Prof. Dr. med. Alexander Zarbock 0251/83-47282
Dr. med. Melanie Meersch 0251/83-47282

Informed Consent Declaration

Prospective, randomized, clinical study for analyzing the influence of the timing of renal replacement therapy on the survival of critically ill patients with acute kidney injury (ELAIN-Trial)

Responsible institution:
Department of Anesthesiology, Intensive Care and Pain Medicine
University of Münster
Albert-Schweitzer-Campus 1, A1
48149 Münster

Principal Investigator: Prof. Dr. med. Alexander Zarbock

________________	________________	________________
(Last name)	(First name)	(Date of birth)

____

(Last name, first name, telephone number of the person obtaining consent)

informed me about the study. I received the patient information related to this trial. I received a copy of the signed statement of consent. I read and understood both documents. I was cleared up on aims, course, chances and risks of the study, my rights and obligations, the insurance cover and the voluntary nature of the participation in this study. I had the possibility to ask questions. These have been sufficiently answered. Additionally, these points have been addressed to:

____
____
____
____
____

It has been explained that during the study period there is an insurance that covers study related harms. I am obligated to contact immediately the study investigators if study related injuries occur.
I am aware that I am able to withdraw my study agreement without giving any reasons (verbally or in writing), without having any disadvantages in my medical treatment.

Data safety

I am aware that for this clinical study personalized data are being raised, especially my medical history and diagnostic findings, for further analysis. The use of the data is subjected to statutory provisions and needs my consent to participate in this study.

1) I agree that the Department of Anesthesiology, Intensive Care and Pain Medicine may record data about my medical condition, on paper as well as on electronic case report forms, for this clinical trial. Where required, data may be transmitted pseudonymously:
    a. In case of adverse events: to the ethics board committee
    b. Further, I agree that authorized and confidential representatives of the university of Münster may take insight to personalized data if this is necessary to prove a proper implementation of the study. For this I release the study investigator from the secrecy obligation.
2) I agree that my data are kept for ten years after termination or completion of the study as law for drug trials prescribes it. Data will be deleted after ten years.

I have been informed that the participation in this trial is voluntary and that I may withdraw my consent at any time of the study period without any disadvantages.

I declare that my participation in the ELAIN-trial is completely voluntary.

I received a copy of the information letter and consent as well as a copy of the general insurance conditions. One copy remains at the trial center.

____

Name of patient

__________ ________________________

Date Signature of patient

Confirmation of person obtaining consent

The patient has been informed about nature, meaning and consequences of the participation in this trial (verbally and in writing) according to the legal regulations of the food and drug legislation. Questions have been sufficiently answered. The patient agreed voluntarily to participate in this trial. A copy of the information letter and consent was delivered to the patient.

____

Name of person obtaining consent

__________ ______________________________

Date Signature of person obtaining consent

Authorized representative - Informed Consent Declaration

Prospective, randomized, clinical study for analyzing the influence of the timing of renal replacement therapy on the survival of critically ill patients with acute kidney injury (ELAIN-Trial)

Responsible institution:
Department of Anesthesiology, Intensive Care and Pain Medicine
University of Münster
Albert-Schweitzer-Campus 1, A1
48149 Münster

Principal Investigator: Prof. Dr. med. Alexander Zarbock

____
(Participant´s name)

I, ______________________________________
(Name of the authorized representative)

have been appointed authorized representative by the above mentioned patient.

I was informed by ______________________________________
(Name of person obtaining consent)

about the study. I received the patient information related to this study and a copy of the signed statement of consent. I read and understood both documents. I was cleared up on aims, course, chances and risks of the study, my rights and obligations, the insurance cover and the voluntary nature of the participation in this study. I had the possibility to ask questions. These have been sufficiently answered. Additionally, these points have been addressed to:

____

____

____

____

It has been explained that during the study period there is an insurance that covers study related harms. I am obligated to contact immediately the study investigators if study related injuries occur.
I am aware that I am able to withdraw my study agreement without giving any reasons (verbally or in writing), without having any disadvantages in my medical treatment.

Data safety

I am aware that for this clinical study personalized data are being raised, especially the medical history and diagnostic findings, for further analysis. The use of the data is subjected to statutory provisions and needs my consent to participate in this study.

1) I agree that the Department of Anesthesiology, Intensive Care and Pain Medicine may record data about the medical condition, on paper as well as on electronic case report forms, for this clinical trial. Where required, data may be transmitted pseudonymously:
    a. In case of adverse events: to the ethics board committee
    b. Further, I agree that authorized and confidential representatives of the university of Münster may take insight to personalized data if this is necessary to prove a proper implementation of the study. For this I release the study investigator from the secrecy obligation.
2) I agree that my data are kept for ten years after termination or completion of the study as law for drug trials prescribes it. Data will be deleted after ten years.

I have been informed that the participation in this trial is voluntary and that I may withdraw my consent at any time of the study period without any disadvantages.

I declare that the above-mentioned patient will participate in the ELAIN Trial.

I received a copy of the information letter and consent as well as a copy of the general insurance conditions. One copy remains at the trial center.

____

Name of patient

____

Name of authorized representative

__________ ______________________________

Date Signature of authorized representative

Confirmation of person obtaining consent

The authorized representative has been informed about the nature, meaning and consequences of the participation in this trial (verbally and in writing) according to the legal regulations of the food and drug legislation. Questions have been sufficiently answered. The authorized representative agreed voluntarily on participation. A copy of the information letter and consent was delivered to the authorized representative.

____

Name of person obtaining consent

__________ ____________________________________

Date Signature of person obtaining consent

Caregiver - Informed Consent Declaration

Prospective, randomized, clinical study for analyzing the influence of the timing of renal replacement therapy on the survival of critically ill patients with acute kidney injury (ELAIN-Trial)

Responsible institution:
Department of Anesthesiology, Intensive Care and Pain Medicine
University of Münster
Albert-Schweitzer-Campus 1, A1
48149 Münster

Principal Investigator: Prof. Dr. med. Alexander Zarbock

____
(Participant´s name)

I, ____am the caregiver of the above mentioned patient.
(Name of the caregiver)

I was informed by ____
(Name of person obtaining consent)

about the study. I received the patient information related to this study and a copy of the signed statement of consent. I read and understood both documents. I was cleared up on aims, course, chances and risks of the study, my rights and obligations, the insurance cover and the voluntary nature of the participation in this study. I had the possibility to ask questions. These have been sufficiently answered. Additionally, these points have been addressed to:

____
____
____
____
____

It has been explained that during the study period there is an insurance that covers study related harms. I am obligated to contact immediately the study investigators if study related injuries occur.
I am aware that I am able to withdraw my study agreement without giving any reasons (verbally or in writing), without having any disadvantages in the medical treatment.

Data safety

I am aware that for this clinical study personalized data are being raised, especially the medical history and diagnostic findings, for further analysis. The use of the data is subjected to statutory provisions and needs my consent to participate in this study.

1) I agree that the Department of Anesthesiology, Intensive Care and Pain Medicine may record data about the medical condition, on paper as well as on electronic case report forms, for this clinical trial. Where required, data may be transmitted pseudonymously:
    a. In case of adverse events: to the ethics board committee
    b. Further, I agree that authorized and confidential representatives of the university of Münster may take insight to personalized data if this is necessary to prove a proper implementation of the study. For this I release the study investigator from the secrecy obligation.
2) I agree that my data are kept for ten years after termination or completion of the study as law for drug trials prescribes it. Data will be deleted after ten years.

I have been informed that the participation in this trial is voluntary and that I may withdraw my consent at any time of the study period without any disadvantages.

I declare that the above-mentioned patient will participate in the ELAIN Trial.

I received a copy of the information letter and consent as well as a copy of the general insurance conditions. One copy remains at the trial center.

____

Name of patient

____

Name of caregiver

____________ ______________________________

Date Signature of caregiver

Confirmation of person obtaining consent

The caregiver has been informed about the nature, meaning and consequences of the participation in this trial (verbally and in writing) according to the legal regulations of the food and drug legislation. Questions have been sufficiently answered. The caregiver agreed voluntarily to participate in this trial. A copy of the information letter and consent was delivered to the caregiver.

____

Name of person obtaining consent

__________ ______________________________

Date Signature of person obtaining consent

Information for the consultant physician

Prospective, randomized, clinical study for analyzing the influence of the timing of renal replacement therapy on the survival of critically ill patients with acute kidney injury (ELAIN-Trial)

Responsible institution:
Department of Anesthesiology, Intensive Care and Pain Medicine
University of Münster
Albert-Schweitzer-Campus 1, A1
48149 Münster

Principal Investigator: Prof. Dr. med. Alexander Zarbock

Declaration of an independent physician about the study participation of the following patient

____
(Name of the patient)

I declare the following:

I examined the patient to see whether she/he is able to understand the nature, meaning and consequences of her/his action and of the clinical trial. The examination showed that the patient is

- o able to give consent
- o unable to give consent

Results and medical explanations for this decision:

____
____
____
____

The study admission represents

- o a personal therapeutic advantage
- o no personal therapeutic advantage

I declare that,

- it was not possible to establish a legal supervision due to the small therapeutic timeframe
- I read and understood the information and agree to the patients inclusion to the study
- I do not have any indication that an inclusion to this study is against the patients wish
- I am not involved in this study, I do not belong to the physicians in charge and I am not part of the responsible institution

____

Place and date

Name of the consultant physician	Signature of the consultant physician

Confirmation of the attending physician

I confirm that due to the emergency situation the patient's informed consent cannot be provided. As soon as a legal representative is available, her/his consent will be obtained. If the patient regains her/his capacity for consent her/his consent will be obtained.

I handed a copy of the information declaration of study inclusion to the consultant physician.

____

Place and date

Name of the attending physician	Signature of the attending physician
