# Supplementary material for: Early versus late initiation of renal replacement therapy in critically ill patients with acute kidney injury (The ELAIN-Trial): study protocol for a randomized controlled trial
Source: Trials. 2016 Mar 18;17:148. doi: 10.1186/s13063-016-1249-9 (PMC4797166; doi:10.1186/s13063-016-1249-9)
Supplement: Additional file 3: — Literature overview for the design of the ELAIN trial. (PDF 68 kb) [file 13063_2016_1249_MOESM3_ESM.pdf]

	Parameters at the time of RRT		
**Study**	**Early RRT**	**Late RRT**	**Outcome**
Bagshaw et al. ; prospective study	Serum creatinine ≤ 309 µmol/l; serum urea ≤ 24.2 mmol/l	Serum creatinine > 309 µmol/l; serum urea > 24.2 mmol/l	Hospital mortality, 71 versus 53.4%, p < 0.00001
Shiao et al. ; prospective study	AKI as per RIFLE classification; no AKI or RIFLE Risk	AKI as per RIFLE classification; RIFLE Injury or Failure	Hospital mortality, 43 versus 75%, p = 0.002
Chou et al.; retrospective study	RIFLE-0 or RIFLE-Risk	RIFLE-Injury or RIFLE-Failure	Hospital mortality, 70.8 versus 69.7 %, p > 0.05
Liu et al.; prospective study	Serum urea < 27.1 mmol/l	Serum urea > 27.1 mmol/l	Hospital mortality, 35 versus 41%, p=0.09
Gettings et al.; retrospective study	Serum urea < 21.4 mmol/l	Serum urea > 21.4 mmol/l	Hospital mortality, 61 versus 80%, p = 0.041
Elahi et al.; retrospective study	Urine output < 100 ml in 8 h	Serum urea ≥ 30 mmol/l or serum creatinine ≥ 250 µmol/l or K^+ > 6 mmol/l	Hospital mortality, 22 versus 43%, p < 0.05
Bouman et al.; RCT	Urine output < 30 ml/h for 6 h and creatinine clearance < 20 ml/min	Serum urea > 40 mmol/l or K^+ > 6.5 mmol/l or severe pulmonary edema	28-day mortality, 29 versus 25%, p = 0.8
Demirkilic et al.; retrospective study	Urine output < 100 ml within 8h post-surgery	Serum creatinine > 440 µmol/l or K^+ > 5.5 mmol/l	Hospital mortality, 23.5 versus 56%, p = 0.016
Sugahara et al.; RCT	Urine output < 30 ml/h for 3h	Urine output < 20 ml/h for 2h	14-day mortality, 14 versus 86%, p < 0.01

Table 1 Literature overview: The ELAIN-trial was planned based on these findings. The mortality rate in the late group was estimated 55%. The reduction of 18% is calculated on the mortality difference between the early and the late group, which results in a difference of 17.9778% favoring the early group.
